# Loss of the receptor tyrosine kinase Axl leads to enhanced inflammation in the CNS and delayed removal of myelin debris during Experimental Autoimmune Encephalomyelitis

**DOI:** 10.1186/1742-2094-8-49

**Published:** 2011-05-15

**Authors:** Jason G Weinger, Celia F Brosnan, Olivier Loudig, Michael F Goldberg, Fernando Macian, Heather A Arnett, Anne L Prieto, Vladislav Tsiperson, Bridget Shafit-Zagardo

**Affiliations:** 1 Department of Pathology, Albert Einstein College of Medicine, 1300 Morris Park Avenue, Bronx, New York 10461, USA; 2 Department of Epidemiology & Population Health, Albert Einstein College of Medicine, 1300 Morris Park Avenue, Bronx, New York 10461, USA; 3 Department of Microbiology & Immunology, Albert Einstein College of Medicine, Bronx, NY 10461, USA; 4 Department of Inflammation, Amgen Inc., 1201 Amgen Court West, Seattle, WA 98119, USA; 5 Department of Psychological and Brain Sciences, Indiana University, 1101 East 10th Street, Bloomington, IN 47405, USA

## Abstract

**Background:**

Axl, together with Tyro3 and Mer, constitute the TAM family of receptor tyrosine kinases. In the nervous system, Axl and its ligand Growth-arrest-specific protein 6 (Gas6) are expressed on multiple cell types. Axl functions in dampening the immune response, regulating cytokine secretion, clearing apoptotic cells and debris, and maintaining cell survival. Axl is upregulated in various disease states, such as in the cuprizone toxicity-induced model of demyelination and in multiple sclerosis (MS) lesions, suggesting that it plays a role in disease pathogenesis. To test for this, we studied the susceptibility of Axl-/- mice to experimental autoimmune encephalomyelitis (EAE), an animal model for multiple sclerosis.

**Methods:**

WT and Axl-/- mice were immunized with myelin oligodendrocyte glycoprotein (MOG)_35-55 _peptide emulsified in complete Freund's adjuvant and injected with pertussis toxin on day 0 and day 2. Mice were monitored daily for clinical signs of disease and analyzed for pathology during the acute phase of disease. Immunological responses were monitored by flow cytometry, cytokine analysis and proliferation assays.

**Results:**

Axl-/- mice had a significantly more severe acute phase of EAE than WT mice. Axl-/- mice had more spinal cord lesions with larger inflammatory cuffs, more demyelination, and more axonal damage than WT mice during EAE. Strikingly, lesions in Axl-/- mice had more intense Oil-Red-O staining indicative of inefficient clearance of myelin debris. Fewer activated microglia/macrophages (Iba1+) were found in and/or surrounding lesions in Axl-/- mice relative to WT mice. In contrast, no significant differences were noted in immune cell responses between naïve and sensitized animals.

**Conclusions:**

These data show that Axl alleviates EAE disease progression and suggests that in EAE Axl functions in the recruitment of microglia/macrophages and in the clearance of debris following demyelination. In addition, these data provide further support that administration of the Axl ligand Gas6 could be therapeutic for immune-mediated demyelinating diseases.

## Background

Axl, a member of the TAM family of receptor tyrosine kinases, has been shown to function in dampening the immune response, regulating cytokine secretion, clearing apoptotic cells and debris, and maintaining cell survival [1-5]. In the nervous system, Axl is widely expressed on microglia, oligodendrocytes, and neurons [4,6-9], and its ligand Growth-arrest-specific protein 6 (Gas6) is expressed on neurons, endothelial cells, astrocytes, and in the CSF [5,10-13]. Although Axl and Gas6 are highly expressed during development, Axl is expressed at low levels in the adult central nervous system (CNS), but is upregulated in disease states, such as in the cuprizone toxicity-induced model of demyelination and in multiple sclerosis (MS) lesions [5,11,14,15].

*In vitro*, Axl signaling can reduce expression of the pro-inflammatory cytokine TNFα by upregulating TWIST, resulting in TWIST binding the E-box of TNFα, thereby blocking NFκB-dependent transcription of TNFα [16,17]. Several cytokines and chemokines including TNFα, and the potent immune cell chemoattractant chemokines MCP1 (CCL2) and RANTES (CCL5) are upregulated in EAE and MS lesions, and contribute to inflammation and demyelination [18-24].

It is well established that inflammation in the CNS can block remyelination but clearance of debris by phagocytosis can allow for efficient remyelination (for review see [25]). Microglia, the resident CNS immune cells of monocyte lineage, are capable of phagocytosis, as well as secreting trophic factors that help repair damage [26-28]. Clearance of apoptotic cells and debris is a critical pathologic response to inflammation. If inflammation is left unchecked it can lead to tissue necrosis and further apoptosis and inflammation. Under pathologic situations, microglia become activated, migrate to the site of injury, surround damaged and dead cells, and clear tissue debris from the area [29]. Axl signaling has a role in phagocytosis; *in vitro *studies have shown that deletion of Axl reduced phagocytosis by fifty percent [30]. Thus, upregulation of Axl in MS lesions may reflect an attempt to protect resident CNS cells from apoptosis, to dampen inappropriate activation of the immune response, and to aid in clearing myelin debris [5].

In this study, we sought to characterize the role of Axl in the CNS following MOG_35-55_-induced EAE, an autoimmune disease of the CNS that shares several clinical and pathologic features with MS, including breakdown of the blood brain barrier (BBB), perivascular inflammation, demyelination, and axonal degeneration. We studied the acute phase of disease in EAE-induced WT and Axl-/- mice. We speculated that Axl is important in minimizing an immune-mediated CNS insult by dampening the inflammatory response, modulating microglia activation, and protecting against axonal damage and demyelination.

## Methods

### Animals

Axl-/- and Tyro3-/- mice were obtained from Dr. Greg Lemke at the Salk Institute and further backcrossed more than six generations onto C57/Bl6J mice (Jackson ImmunoResearch Laboratories, Bar Harbor, ME) in our select pathogen-free barrier facility. C57/Bl6J WT littermates were used as controls. All experiments were performed with age and sex-matched mice at 8 weeks of age. All protocols followed internationally recognized guidelines and were approved by the Animal Care and Use Committee at the Albert Einstein College of Medicine, Protocol Number 20080308; NIH OLAW/NIH Assurance number is A3312-01.

### MOG-induced EAE

Mice were immunized with MOG peptide_35-55 _(3 mg/ml; Celtek Bioscience, Franklin, TN) emulsified in an equal volume of CFA composed of *mycobacterium tuberculosis *(10 mg/ml; Difco Laboratories, Detroit, MI) in incomplete Freund's adjuvant (Difco Laboratories). Mice were anaesthetized with isoflurane and 100 μl of emulsion was injected subcutaneously on each flank (200 μl total/mouse) on day 0. In addition, 200 μl of pertussis toxin (2.5 μg/ml; List Biological Laboratories, Campbell, CA) was injected into the tail vein on days 0 and 2. In our colony, this regimen produced a milder form of EAE than has been reported in the literature [31]. Mice were monitored and graded daily for clinical signs of disease as follows: 0, no disease; 1, limp tail; 2, limp tail and hind limb weakness; 3, hind limb paralysis; 4, hind limb and front limb paralysis; 5, moribund. Mice that were considered borderline between scores were given a half score; 0.5, 1.5, 2.5, 3.5. Data analyses shown in all figures were performed during the acute phase of disease, days 14-19.

### Spinal cord dissection and tissue preparation

Mice were anaesthetized with ethyl ether (Fisher Scientific, Pittsburgh, PA) and sacrificed by total body perfusion with 4% paraformaldehyde (Fisher Scientific), Trump's Fixative (4% gluteraldehyde (Polysciences Inc, Warrington, PA) + 2% paraformaldehyde), or ice cold 1× PBS, pH 7.3. Spinal cords were removed and dissected into cervical, thoracic, and lumbar regions. Trump fixed tissue was embedded in epon and one micron thick sections were stained with toluidine blue. For immunohistochemistry, paraformaldehyde fixed tissue was immersed in 30% sucrose overnight at 4°C prior to embedding in paraffin or OCT. Representative images shown are WT CI = 1.5 and Axl-/- CI = 2.5, equivalent to the average CI for each genotype at the peak of acute phase of disease. For protein or RNA isolation, the spinal cord was flushed out using an 18 gauge needle and 10 cc syringe with ice cold 1× PBS. For total protein homogenates, PBS-perfused tissue was sonicated on ice with a tissue master 125 sonicator (Omni International, Marietta, GA) in protein buffer (140 mM NaCl, 1 mM Tris, pH 7.4) containing 0.5% Triton X-100 and protease inhibitors [2 μg/mL leupeptin; 2 mM ethylene glycol-bis(β-aminoethylether)-N,N,N',N'-tetraacetic acid; 4 μg/mL pepstatin; 5 mM sodium pyrophosphate; 30 mM β-glycerophosphate; 30 mM sodium fluoride; 100 mM sodium orthovanadate; 100 mM 4-(2-aminoethyl) benzenesulfonyl fluoride hydrochloride]. The homogenates were cleared by centrifugation at 4°C at 7000 g for 10 min and aliquots were frozen at -80°C. For RNA isolation, lumbar sections of spinal cord were sonicated in Qiazol and isolated with an RNeasy Lipid Tissue Mini Kit (Qiagen Inc, Valencia, CA). The RNA samples were quantified on a nanodrop ND-1000 spectrophotometer (Thermo Scientific, West Palm Beach, FL) and aliquoted at 50 ng/μl.

### Western blot analysis

Eighty μg of protein was loaded in 1× final concentration loading buffer containing 2% SDS, 0.017% bromophenol blue dye, and 0.28 M β-mercaptoethanol (loading dye), and separated in a 10% SDS-PAGE [32]. Following electrophoresis, proteins were transferred to nitrocellulose [33], and incubated with 5% non-fat dry milk and 5% goat serum in 1× Tris buffered saline pH 7.4 (1× TBS) for 1 hr at room temperature [34]. After blocking, membranes were incubated with respective primary antibodies followed by HRP-conjugated secondary antibodies. Primary monoclonal antibodies (mAbs) and polyclonal antibodies (pAbs) included: β-actin mAb (IgG2A, 1:5,000; Sigma) and Axl pAb (1:1000). Secondary antibodies (Jackson ImmunoResearch Laboratories, West Grove, PA) included: goat α-rabbit IgG (1:10,000), and goat α-mouse IgG2A (1:10,000). Visualization of all secondary antibodies was by electrochemiluminescence (ECL) (GE Healthcare, Piscataway, NJ). Relative densitometric intensity (rdi) was determined for each band as previously described [5].

### Ex vivo proliferation assay

Axl-/- and WT mice were sensitized with MOG_35-55 _as described above and lymphocytes were harvested from draining lymph nodes on day 16. Single cell suspensions were prepared and cultured *in vitro *with 20 μg/ml MOG_35-55 _peptide. After 3 days, ^3^H-thymidine was added to the cultures for an additional 48 h and assayed for thymidine uptake using a cell harvester [35].

### Quantitative RT-PCR

The Taqman RNA-to-C_T _1-Step kit (Applied Biosystems, Foster City, CA) was used with a total of 100 ng of RNA per reaction with TNFα, MCP1, RANTES, and GAPDH primers (Applied Biosystems). All results were normalized to GAPDH as an internal control. The reactions were aliquoted in triplicate in an optical 96 well-plate; RNase-free water was used as a blank. Reactions were run in the StepOnePlus real-time PCR machine (Applied Biosystems) with the following cycles: hold stage, 48°C for 15 min for reverse transcription, 95°C for 10 min to activate the DNA polymerase, followed by 40 cycles at 95°C for 15 seconds, and 60°C for 1 min. The real-time quantification was monitored directly by the StepOnePlus software and the comparative thresholds (C_T_) were identified for each gene with each RNA sample and calculated at the end of the measurements. After normalization of the (C_T_), the fold change was determined as fold change = 2^-(ΔΔC^T^) ^where ΔΔC_T _is defined as the normalized change in C_T _between two groups. Statistics were performed on the normalized C_T _values.

### Immunohistochemistry

Paraffin-embedded and frozen sections were prepared from the paraformaldehyde fixed tissue. Seven micron paraffin-embedded sections were deparaffinized, rehydrated and then incubated for 30 min with 1× TBS containing 0.25% Triton X-100 (0.01% Triton X-100 for Axl pAb) plus 3% hydrogen peroxide, followed by a 1 hr incubation in 5% goat serum and 5% nonfat dry milk in 1× TBS, and incubated with antibodies diluted in 5% nonfat dry milk in 1× TBS, overnight at 4°C, with the exception of CD45 and CD3, which were not incubated in 0.25% Triton X-100 plus 3% hydrogen peroxide. Primary antibodies consisted of mAb to MBP (SMI99; 1:1500; Covance, Inc, Emeryville, CA), mAb CD45 (1:20; BD Biosciences, San Jose, CA), or mAb Neurofilament H nonphosphorylated (SMI32; 1:30,000; Covance). Ten micron frozen sections were stained with the mAb MAC3 (1:20; BD Pharmingen), pAb Iba1 (1:400; Wako Chemicals, Richmond, VA) or pAb Axl (1:200; Amgen, Thousand Oaks, CA). Control staining consisted of omission of the primary antibody or for a polyclonal antibody control preimmune serum was used. Sections were washed 3× in 1× TBS and incubated with mouse monoclonal or rabbit polyclonal secondary antibodies followed by incubation with the appropriate Vecta staining kit (Vector Laboratories, Inc., Burlingame, CA) and visualized by 0.3 mg/ml DAB (Sigma) in 0.1 M TRIS pH 7.4 plus 0.009% hydrogen peroxide. Quantification was determined using an objective stage micrometer with a reticule (Nikon, Melville, NY). Quantification of CD45 by integrated density was performed in Image J and statistical analysis was evaluated in Prism.

### Immunofluorescence

Ten micron thick frozen sections were prepared from spinal cords from 4% paraformaldehyde perfused mice. Sections placed on Excell plus slides (Richard-Allan Scientific, Kalamazoo, MI) were dried by placing them at 37°C for 2 hr, or overnight at room temperature. Sections were washed in 1× TBS and blocked with 5% milk in 1× TBS plus 5% goat serum for 1 hr at room temperature. Double-label immunofluorescence was performed sequentially. Between all primary and secondary antibody incubation steps sections were washed 3× with 1× TBS. Primary antibody: CD3 hamster mAb (1:200, Novus Biological, Littleton, CO) overnight at 4°C, secondary antibody: goat anti-hamster Alexa-568 (1:1000, Invitrogen, Carlsbad, CA) for 1 hr at room temperature, followed by Iba1 pAb (1:400, Wako Chemicals) overnight at 4°C, secondary antibody: goat anti-rabbit Alexa-633 (1:1000, Invitrogen) for 1 hr at room temperature. All antibodies were diluted in 5% milk in 1× TBS. Hoechst stain (1:1000; Sigma), 10 min at room temperature, was used as a counterstain for nuclei. Sections were mounted with Aquamount (Biomeda, Foster City, CA). All samples were examined under an Olympus (Melville, NY) 1 × 70 with a 20× numerical aperture 1.0 or a 40× numerical aperture 1.0 Plan Apo optics inverted microscope with a Photometrics (Roper Scientific, Tucson, AZ) Censys-cooled CCD camera; images were collected with IPLab Spectrum software (Scanalytics, Fairfax, VA).

### Oil-Red-O staining

Frozen sections were incubated in distilled water for 1 min followed by a 2 min incubation in 100% propylene glycol (Polyscientific, Bayshore, NY) whereupon the sections were transferred to Oil-Red-O for 36 h at room temperature. Sections were incubated for 1 min in 85% propylene glycol, rinsed briefly with distilled water, lightly stained with hematoxylin and mounted with gelatin mounting medium.

### Serum Protein Analysis

Individual serum samples from naïve wild-type (n = 6) and Axl-/- (n = 5) mice, as well as MOG-peptide induced EAE wild-type (n = 5) and Axl-/- (n = 9) mice, underwent quantitative proteomic analysis for 60 chemokines, growth factors and cytokines as previously described (Rules Based Medicine, Inc., Austin, TX) [36]. Briefly, sera were analyzed in a blinded fashion at a previously optimized dilution in a Luminex-based platform. Each antigen assay was calibrated with standard curves, performed in duplicate, and intensity measurements were interpreted as protein concentrations within Rules Based Medicine's proprietary software.

### CNS Preparation for Flow Cytometry and Analysis

All experimental data was acquired on an LSR II flow cytometer (BD Biosciences). Data analysis was performed using FlowJo software (Tree Star, Ashland, OR). Following dissection of brain and spinal cord, the organs were injected with 2 ml of RPMI1640 containing 0.3 Wünsch Units/ml Liberase TL (Roche Molecular Biochemicals, Indianapolis, IN) and 10 μg/ml DNAse I (Sigma, St. Louis, MO) and incubated at 37°C for 30 minutes. The organs were then mashed through a 70 micron cell strainer, washed twice with RPMI and centrifuged at 400 × g for 10 min at 4°C. The CNS pellets were then resuspended in 30% percoll and overlayed onto 70% percoll, followed by centrifugation at 600 × g for 25 min at room temperature. Cells at the interface were collected as the CNS mononuclear cell fraction, and analyzed by flow cytometry. Briefly, cells were resuspended in flow cytometry buffer (PBS + 2% Fetal Calf Serum + 0.05% NaN_3_) and blocked with rat anti-mouse FcγII/III (clone 2.4G2) and stained with a cocktail of the following antibodies; CD4 (clone GK1.5), CD8 (clone 53-6.7), CD3e (clone 145-2C11), CD11b (clone M1/70), CD11c (N418), Ly6G (clone 1A8) (BD Biosciences), CD45 (clone 30-F11) and NKp46 (clone 29A1.4) (eBioscience, San Diego, CA) for the evaluation of CNS infiltrating leukocyte populations. For regulatory T cell (T regs) analysis, CNS preparations were stained with BluVID, blocked with 2.4G2 and surface stained with anti-CD3, anti-CD4, and anti-CD25 (clone PC-61; BD Biosciences). Cells were then prepared for intracellular staining using a FoxP3 staining kit (eBioscience) according to the manufacturer's instructions and stained with anti-FoxP3 (clone FKJ-16S) (eBioscience).

### Intracellular Cytokine Staining

For analysis of cytokine producing CD4^+ ^T cells subsets, CNS mononuclear cells were prepared from WT and Axl-/- mice 2 weeks following MOG induction of EAE and re-stimulated with 10 μg/ml MOG_35-55 _peptide plus 1 μg/ml soluble anti-CD28 mAb (eBioscience). After incubation (37°C, 5% CO_2_) for 1 h, 10 μg/ml brefeldin A and 2 μM monensin (Sigma-Aldrich) was added for an additional 4 h. Cells were labeled with BluVID, blocked with mAb 2.4G2, and stained with antibodies against CD3e, CD4, CD11b, and CD45R/B220 (clone RA3-6B2) (BD Biosciences). Cells were fixed with 2% paraformaldehyde, washed with permeabilization buffer (1× PBS with 1 mM Ca^2+^, 1 mM Mg^2+^, 1 mM HEPES, 2% FCS, and 0.1% saponin), and then blocked in permeabilization buffer plus 5% normal mouse serum (Jackson ImmunoResearch Laboratories). Intracellular cytokines were detected with antibodies against IL-2 (clone JES6-5H4), IL-17A (clone eBio17B7) (eBioscience), IFN-γ (clone XMG1.2), and TNFα (MP6-XT22) (BD Biosciences) [37].

### Quantitative Procedures and Statistical Analysis

A student's t-test was performed for clinical indices of WT and Axl-/- mice derived from 3 independent experiments. Inflammatory cuffs were quantified using a minimum of 4 sections per cord from 3 WT and 4 Axl-/- mice. For toluidine-blue stained sections, lesions were measured (mm^2^) using a reticule and a micrometer bar inserted into the ocular of a Nikon microscope. Statistical analysis (student t-test) was performed using mice from the same experiment and time point. Iba1+ activated microglia were counted in and surrounding 10 lesions in multiple sections from WT and Axl-/- mice. SMI32+ neurofilaments were counted in the ventral and dorsal funiculi of 3 sections per mouse for 3 WT and 3 Axl-/- mice. Statistical analysis was obtained by a student's t-test.

## Results

### Clinical expression of EAE in Axl-/- mice

**Figure 1:**
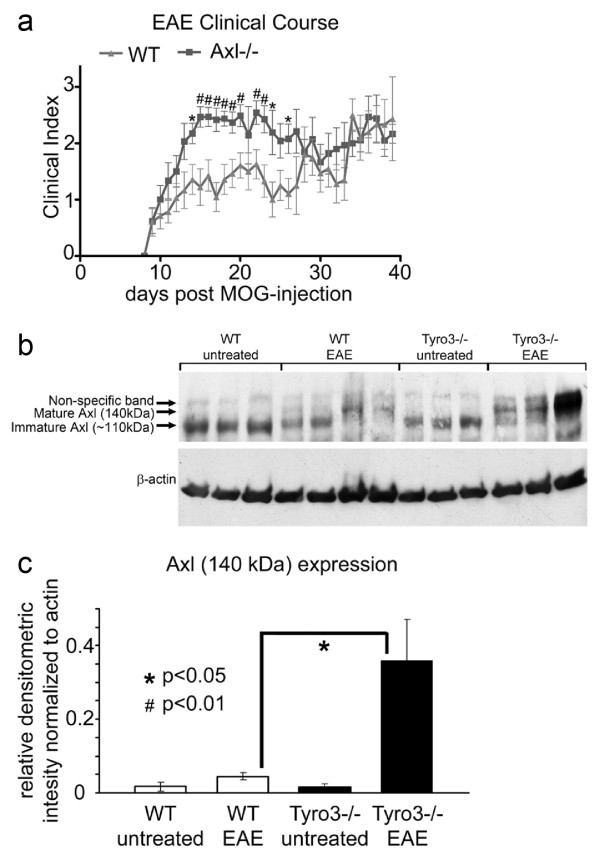
**Axl-/- mice have a more severe clinical course than WT mice during MOG-induced EAE**. a) EAE clinical course for Axl-/- and WT female mice. Three experiments were combined: WT (n = 15) and Axl-/- (n = 21) mice. All mice were housed in Barrier. b) Western blot analysis was performed using an Axl pAb on 80 μg of WT naïve (untreated), WT EAE, Tyro3-/- naïve (untreated), and Tyro3-/- EAE lumbar spinal cord homogenates. The mature Axl band migrates at 140 kDa, and the immature band (lacking post translational modifications) migrates at approximately 110 kDa. N = 3 for all groups except WT EAE where n = 4. c) The relative densitometric intensity (rdi) was determined for each band and normalized to β-actin. The average values for 140 kDa Axl are shown. * = p < 0.05, # = p < 0.01.

Following sensitization with MOG peptide, Axl-/- female mice (n = 21) had significantly higher clinical indices than WT mice (n = 15) between days 14 and 26, which included the acute phase and beginning of the chronic phase of the disease (Figure 1a: 3 combined experiments; d14, 24, 26: p < 0.05; d15-20, 22, 23: p < 0.01; d21, 25: not significant; p > 0.05). The end of the acute phase and beginning of the chronic disease phase occurred at day 23. There was no statistical difference between the two treated groups prior to day 14 or during the chronic phase of EAE, after day 26. Each of the individual experiments showed significant differences in the acute phase between WT and Axl-/- mice.

Concurrent experiments using TAM family member Tyro3 knockout mice (Tyro3-/-) showed no significant difference in clinical scores between WT and Tyro3-/- mice during the acute or chronic phases of disease (data not shown). Studies in other receptor tyrosine kinase families have shown that related receptor tyrosine kinases may be upregulated to compensate for the loss of another receptor [38]. Thus, we examined whether the lack of increased disease severity in Tyro3-/- was a result of compensation by an upregulation of the Axl receptor, using Western blot analysis of lumbar spinal cord from naïve and MOG-sensitized WT or Tyro3-/- mice (Figure 1b,c). In naïve WT and Tyro3-/- mice, there was no difference in the expression of either the immature (110 kDa) or mature form of Axl, which migrates at 140 kDa [39]. In WT EAE mice, the 140 kDa Axl was increased 2.7-fold over naïve WT mice. However, the 140 kDa form of Axl was increased 8-fold in Tyro3-/- EAE mice over WT EAE mice (Figure 1c, p = 0.02). Furthermore, there was a visible decrease in the amount of the smaller immature form of Axl in spinal cord homogenates from Tyro3-/- EAE mice, presumably because it was converted to the mature 140 kDa form. From these data, we conclude that activation of Axl occurs in the CNS of animals with EAE and that upregulation of Axl in Tyro3-/- mice likely compensates for loss of this TAM family receptor.

### Analysis of peripheral immunological parameters in Axl-/- mice sensitized for EAE

Although Axl is not expressed on T cells, to verify that the loss of Axl did not affect the peripheral immune response to antigen, cytokine profiles of sera from naïve and sensitized mice were examined, using an automated assay (see methods). No significant difference in values for 60 different factors (see methods) was detected in naïve or EAE-sensitized WT or Axl-/- mouse sera. In both WT and Axl-/- mice sensitized for EAE, increased expression over that found in naïve mice was noted for KC/Groα, MCP-1, MMP-3, MIP1γ, MIP1α, M1P1β, MIP2, MIP3β, IL-4, IL-6, IL-7, IL-11, VEGF, LIF, OSM, and TNFα. To explore the possibility that there was differential activation of peripheral lymphocytes in the Axl-/- mice during EAE lymphocytes were isolated from draining lymph nodes on day 16 post-MOG injection. Following activation with 20 μg/ml MOG, the fold-increase for proliferating lymphocytes was 3.9 and 4.1 for WT and Axl-/- mice, respectively (data not shown). Thus, there was no indication that the loss of Axl affected the peripheral immune response to MOG in these mice.

### Analysis of immunological parameters in the CNS of Axl-/- mice sensitized for EAE

#### Number and cellular-makeup of inflammatory infiltrates consistent between Axl-/- and WT mice

**Figure 2:**
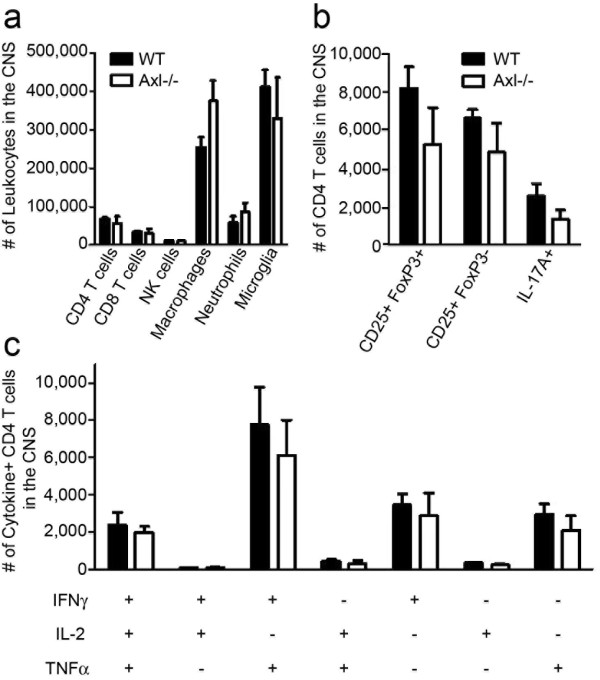
**No difference in the number of leukocytes or cytokine expression from CD4+ T cells in the CNS**. Mononuclear cell preparations from brain and spinal cord were stained for flow cytometric analysis to detect differences in the phenotype of cellular infiltrates (a). The gating strategy allowed us to discriminate populations of microglia, macrophages, neutrophils, NK cells, CD4 and CD8 T cells (a) as well as CD4 T cell subsets (b). CD4 T cells were restimulated with MOG_35-55 _peptide and analyzed for cytokine production (c). Multi-parametric flow cytometric analysis was performed on polyfunctional CD4 T cell subsets capable of producing 3, 2, or 1 cytokine(s) simultaneously. Student's t-test revealed no significant differences between WT and Axl-/- mice. These data are representative of two experiments each performed in triplicate, with 4 mice/group/experiment at day 19.

To determine the cellular makeup of inflammatory infiltrates in the CNS, flow cytometric analysis was performed on cells isolated from the brains and spinal cords of Axl-/- and WT mice sensitized for EAE (Figure 2). The leukocyte population was identified by staining for CD45. As shown in Figure 2, no significant difference in the number of infiltrating leukocytes (CD4 or CD8 T cells, NK cells, macrophages, neutrophils, microglia)(Figure 2a), CD4+ T cell subsets (CD4+/FoxP3+ Treg, CD25+/FoxP3-, MOG-specific IL-17A+) (Figure 2b), or cytokine positive CD4+ T cells (IFN-γ, IL-2, TNFα) (Figure 2c) was detected.

### Pro-inflammatory cytokines are upregulated in Axl-/- mice during EAE

**Figure 3:**
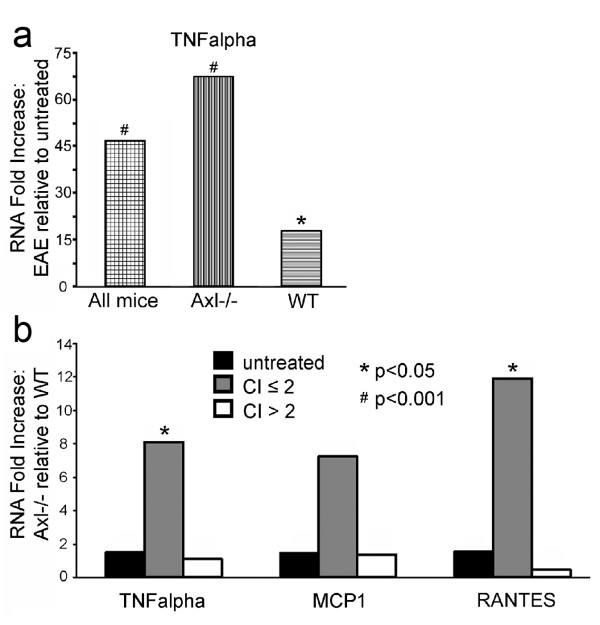
**Transcripts for pro-inflammatory cytokine/chemokines are increased in the lumbar spinal cord of Axl-/- mice relative to WT during the acute phase of EAE**. a) 45.7-fold increase of TNFα transcript in Axl-/- and WT lumbar spinal cord (combined) during the acute phase of EAE relative to naïve Axl-/- and WT mice (combined); 68.8-fold increase in Axl-/- EAE mice relative to untreated Axl-/-; 18.7-fold increase in WT EAE mice relative to WT untreated mice. b) Increased TNFα, MCP1 and RANTES transcripts were found in Axl-/- lumbar spinal cord compared to WT. Samples of RNA from lumbar spinal cord were grouped as untreated (non-MOG EAE-induced), mice with CI's ≤ 2, or mice with CI's > 2. The fold increase of pro-inflammatory cytokine/chemokines in Axl-/- lumbar spinal cord relative to WT lumbar spinal cord was determined. Fold increase for CI ≤ 2; TNFα = 8.1, MCP1 = 7.2, RANTES = 11.9. * p < 0.05, # p < 0.001.

The experiment described above analyzed cytokine production in T cell sub-populations. To further explore the role of Axl in regulating cytokine production in the spinal cord, qRT-PCR was performed. We analyzed lumbar spinal cord at day 16 post-EAE induction to determine whether the loss of Axl resulted in altered pro-inflammatory cytokine and chemokine mRNA expression compared to WT mice during acute EAE. Since it had been reported that Axl regulates TNFα transcription through induction of TWIST, we analyzed TWIST expression but found no difference in the amount of TWIST RNA between naïve and EAE-induced Axl-/- and WT mice (data not shown). Additionally, in naïve Axl-/- and WT lumbar spinal cord there was no difference in TNFα, MCP1, or RANTES mRNA expression. However, as expected the pro-inflammatory cytokines and chemokines TNFα, MCP1, and RANTES were upregulated in the spinal cord during acute EAE. The levels of TNFα mRNA relative to naïve mice in both WT and Axl-/- mice were increased approximately 46-fold (Figure 3a). Individually, Axl-/- mice with EAE had a 68.6-fold increase in TNFα relative to untreated Axl-/- mice and WT mice with EAE had a 18.7-fold increase in TNFα relative to untreated WT mice. When Axl-/- and WT mice with EAE were grouped as severely affected (CI > 2) there was no difference in TNFα, MCP1, or RANTES mRNA. However, in mildly-moderately affected mice with EAE (CI ≤ 2), we found that TNFα (8.1-fold, p < 0.05), MCP1 (7.2-fold, not significant), and RANTES (11.9-fold, p < 0.05) were increased in Axl-/- lumbar spinal cord relative to WT (Figure 3b). While the increase in MCP1 was not significant, it was significant when only mildly affected mice (CI ≤ 1.5) were grouped (12.0-fold, p < 0.05, data not shown).

### Pathological analysis of the spinal cord in mice with EAE

#### Distribution of infiltrating lymphocytes is altered in Axl-/- mice

**Figure 4:**
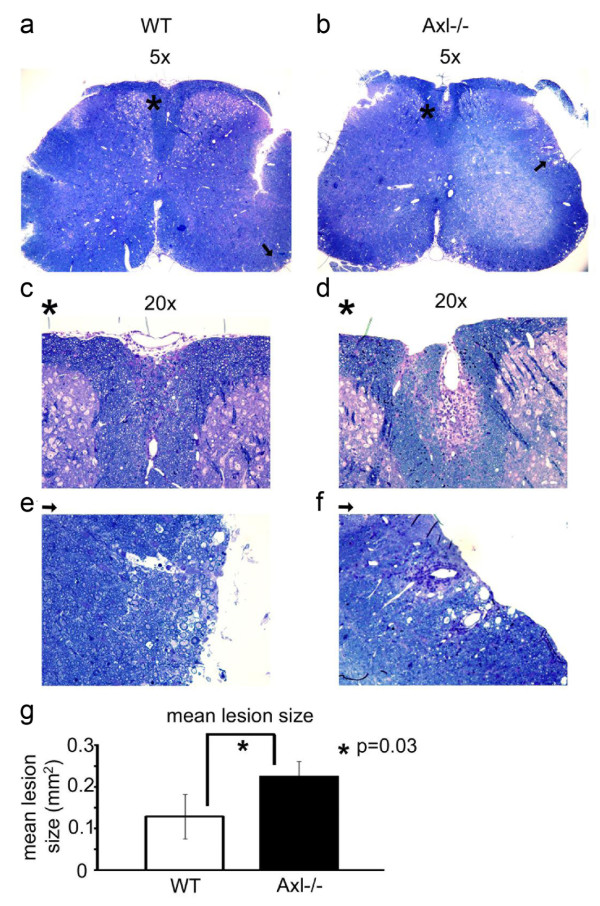
**Axl-/- mice have larger inflammatory cuffs and increased demyelination in the spinal cord than WT mice**. One micron Trump's fixed cross-sections of WT (a,c,e) and Axl-/- (b,d,f) spinal cords were stained with toluidine blue. a,b) Representative 5× sections of whole spinal cord. Two 20× sections with areas of infiltration and demyelination are shown for each 5× section (WT: c,e; Axl-/-: d,f). The 20× sections are labeled with an asterisk (dorsal funiculus; c,d) or arrow (lateral funiculus; e,f) denoting the corresponding area on the 5× section. g) quantitative data for the mean lesion size (mm^2^), see methods.  Ratio of CD3+ to Iba1+ cells per lesion.

To determine the effect of the loss of Axl on the pathological expression of disease, spinal cord sections were prepared from WT (Figure 4a,b,c) and Axl-/- (Figure 4d,e,f) mice during the acute phase of disease, when significant differences in clinical indices were noted. Despite flow cytometry data showing no difference in the number of spinal cord leukocytes between Axl-/- and WT mice, toluidine blue stained sections of Axl-/- spinal cords showed marked infiltration and inflammatory cuffs. The inflammatory cells invaginated the white matter extending toward grey matter, and were associated with myelin loss (Figure 4 d,e,f). In contrast, toluidine blue staining of spinal cords from WT mice showed smaller inflammatory cuffs and less severe myelin loss associated with the cuffs (Figure 4a,b,c). Quantification of the lesion sizes showed lesions in Axl-/- mice (0.23 ± 0.017 mm^2^) were significantly larger than lesions in WT mice (0.139 ± 0.031 mm^2^, p = 0.03, Figure 4g).

**Figure 5:**
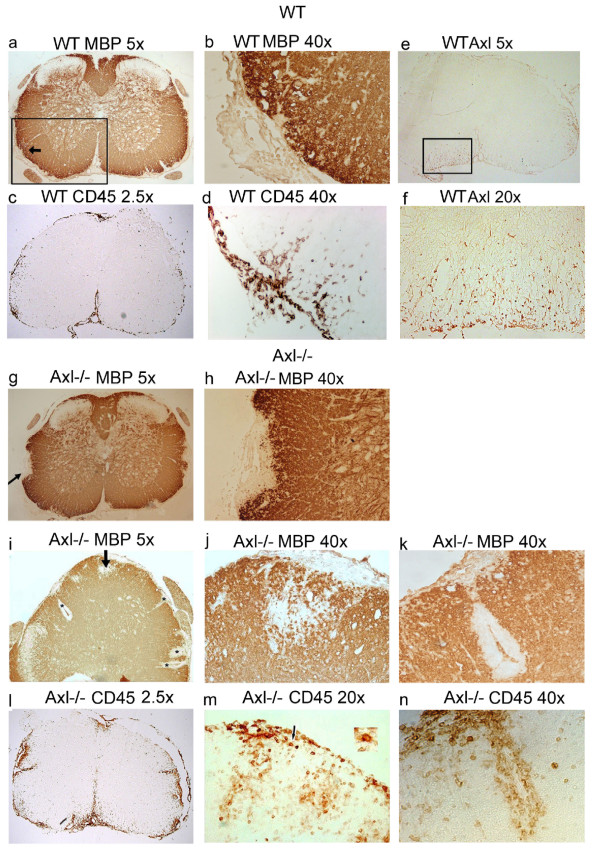
**Demyelination and infiltration of CD45+ cells in spinal cords of Axl-/- mice are enhanced relative to WT mice**. Seven micron paraffin-embedded day 14 WT and Axl-/- spinal cord sections were stained with MBP or CD45 mAb's, and frozen sections were stained with an Axl pAb, and were visualized by DAB. Representative images of WT (a,b) and Axl-/- (g,h,i,j,k) spinal cord stained for MBP. Arrows in a,g,i denote areas of demyelination shown at 40× in b,h,j, respectively. Asterisks in i denote demyelinated lesions; higher magnification of one lesion at 40× is shown in k. e,f) images of a WT mouse stained for Axl; the boxed area in e is shown at 20× in f. c,l) low magnification images of WT (c) and Axl-/- (l) mice stained with CD45. d) serial section of b stained for CD45. Serial sections of j,k stained for CD45 are shown in m,n.

Immunostaining was performed on lumbar and cervical sections of spinal cord, the regions of spinal cord historically shown to be most affected by MOG-induced EAE. To visualize the location of infiltrating leukocytes and their correspondence to areas of demyelination, sequential WT and Axl-/- spinal cord sections were stained with an antibody against MBP (Figure 5a,b,g,h,i,j,k) and CD45 (Figure 5c,d,l,m,n). CD45 staining showed infiltrating leukocytes were distributed differently in the WT and Axl-/- spinal cord. In WT mice, most of the CD45+ cells were at the lateral edges of the spinal cord, consistent with the sparsity of inflammatory cuffs seen in the toluidine blue stained sections (Figure 5c,d). In Axl-/- mice, infiltrating leukocytes were located in the white matter, in inflammatory cuffs (Figure 5 l,m,n). There was a significant increase in CD45 staining in the lumbar spinal cord of Axl-/- mice (n = 4) relative to WT mice (n = 3) (p = 0.01), as determined by relative integrated density. The areas of demyelination, shown by loss of immunoreactivity to MBP, corresponded to the location of infiltrating CD45+ leukocytes (WT: Figure 5b,d; Axl-/-: Figure 5j,k,m,n) confirming the results obtained with toluidine blue staining. Spinal cord sections from WT mice stained with an Axl antibody show that Axl was evident in inflamed white matter (Figure 5e, higher magnification shown in 5f). No Axl staining was seen in EAE-induced Axl-/- mice (data not shown).

#### Fewer microglia/macrophages are present at lesions in Axl-/- mice

**Figure 6:**
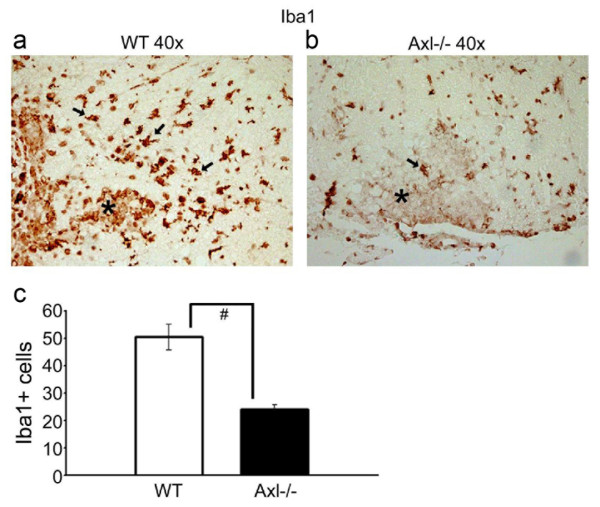
**Axl-/- mice have significantly fewer Iba1+ microglia/macrophages within and surrounding lesions than WT mice**. Ten micron frozen WT and Axl-/- spinal cord sections were stained with an Iba1 pAb. Representative 40× images for WT (a) and Axl-/- (b) mice are shown. The arrows point to Iba1+ microglia/macrophages and the asterisks denote lesion area. c) Iba1+ activated microglia were counted in and surrounding multiple lesions in multiple mice for WT and Axl-/- mice. Statistical analysis was performed; student's t-test confirmed significance between WT and Axl-/- mice; p < 0.01.

**Figure 7:**
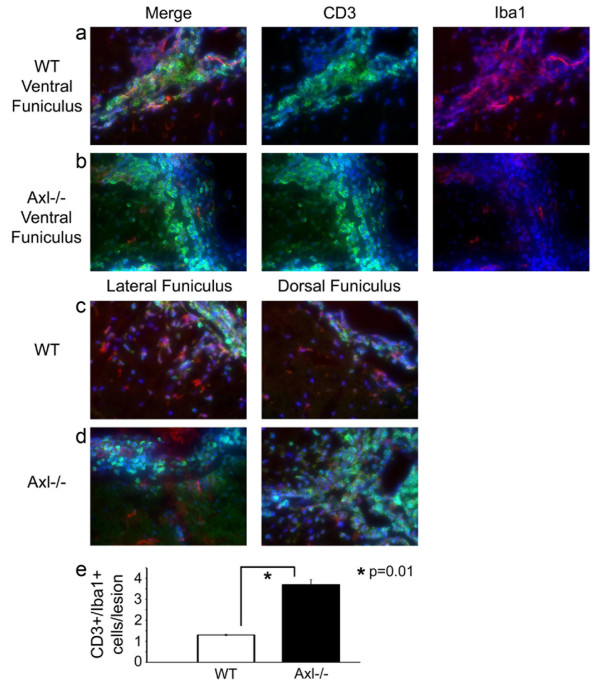
**Double-label immunofluorescence confirm Axl-/- (b,d) mice have fewer Iba1+ microglia/macrophages than WT (a,c) mice in ventral (a,b), lateral and dorsal funiculi (c,d)**. Ten micron frozen spinal cord sections were stained with an Iba1 pAb (red), a CD3 mAb (green), counterstained with Hoechst stain (blue), and visualized by immunofluorescence. a,b) Left panel shows merged triple fluorescence, middle panel shows CD3/Hoechst, and right panel shows Iba1/Hoechst for the ventral funiculus of WT (a) and Axl-/- (b) spinal cords. c,d) Merged images of lateral and dorsal funiculi for WT and Axl-/- spinal cords. All images are 40×. e) CD3+/Iba1+ cells/lesion for WT and Axl-/- mice.

To assess the state of activation of microglia (and macrophages) in the spinal cord, we stained sections for Iba1 (ionized calcium binding adaptor molecule 1). Iba1 is a 17 kDa EF hand protein specifically expressed in microglia/macrophages and is upregulated during cell activation (Figure 6). The results showed that although the overall numbers of Iba1+ cells were the same in Axl-/- and WT spinal cord (data not shown), there were significantly fewer Iba1+ activated microglia and macrophages within and directly surrounding lesions in Axl-/- spinal cord (24.2 ± 1.6) compared to lesions in WT spinal cord (50.3 ± 4.7) as quantified in multiple 40× fields (p < 0.0001; Figure 6c). To better compare the inflammatory cells in and surrounding lesions in Axl-/- and WT mice and to establish that the lesions in Axl-/- mice had fewer activated microglia/macrophages relative to lymphocytes than the lesions in WT mice, double-label immunofluorescence was performed. Antibodies to CD3 (T cells, green) and Iba1 (microglia/macrophages, red) (Figure 7) were used on tissue obtained from the most severely affected mice from each group (CI's > 2). As shown in Figure 7b, in Axl-/- mice, the ventral funiculus, which contains the corticospinal and vestibuolspinal tracts involved in motor control of the limbs and balance, had lesions with a larger ratio of CD3+ T cells (green, left panel merge (Figure 7a,b) and middle panel single (Figure 7a,b)) to Iba1+ activated microglia/macrophages (red, left panel merge (Figure 7a,b) and right panel single (Figure 7a,b)). Furthermore, in WT mice, the microglia/macrophages appeared to tightly encompass areas of infiltration (Figure 7a). Similar results were found in the lateral and dorsal funiculi; compare Figure 7c,d. Thus, although the overall mean number of microglia and macrophages were equivalent in the WT and Axl-/- CNS, as determined by flow cytometry (Figure 2a), there were fewer Iba1+ activated microglia and macrophages within and surrounding lesions in the Axl-/- spinal cord (Figure 7e; p = 0.01).

#### Axl-/- mice have more myelin debris than WT mice

**Figure 8:**
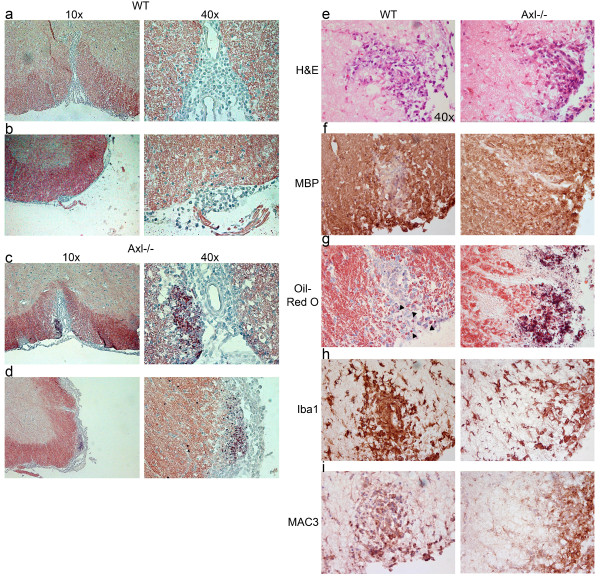
**Axl-/- mice do not clear myelin debris as efficiently as WT mice**. Ten micron frozen WT and Axl-/- spinal cord sections were stained with the lipid dye Oil-Red-O (a,b,c,d,g). Myelin debris appears as dense red clumps. Arrowheads in g show sparse Oil-Red-O+ deposits in the WT lesion. Representative 10× (a,b,c,d left panel) and corresponding 40× (a,b,c,d right panel) images are shown for the ventral (a,c) and lateral (b,d) funiculi of WT (a,b) and Axl-/- mice (c,d, e,f,g,h,i) 40× images of sections showing H+E (e), MBP (f), Iba1 (h) and MAC3 (i) for WT and Axl-/- mice.

Axl has also been implicated in the phagocytic activity of macrophages. To determine if there was more myelin debris in Axl-/- mice, Oil-Red-O staining was performed to detect the presence of lipid debris present in and around lesions. Minimal to no Oil-Red-O+ clumps were present in and around lesions in WT mice (Figure 8a,b); however, there was visible clumping of Oil-Red-O+ material in and around lesions in Axl-/- mice (Figure 8c,d). Myelin lipid debris was present in white matter lesions of the ventral funiculus (Figure 8a,c), lateral funiculus (Figure 8b,d), and dorsal funiculus (data not shown). This suggested that lipid debris from demyelinated axons were efficiently removed from lesions in WT but not Axl-/- mice. We hypothesized that the extensive Oil-Red-O+ material and minimal surrounding Iba1+ microglia/macrophages during the acute phase of EAE in Axl-/- mice may be due to a lag in migration of these cells to sites of inflammation. To verify that the fewer number of cells was not due to a loss of Iba1 expression in the Axl-/- mice we studied a second microglia/macrophage marker, MAC3. By H+E, we selected comparable lesions from WT and Axl-/- spinal cord to evaluate MBP, Oil-Red-O, Iba1, and MAC3 staining (Figure 8e,f,g,h,i). Again, we observed extensive Oil-Red-O+ debris in the Axl-/- spinal cord, and minimal debris in WT mouse (arrowheads 8 g). Additionally, the Oil-Red-O+ debris in the lumbar spinal cord of WT mice was consistently surrounded by a concentric ring of Iba1+ and MAC3+ inflammatory cells (Figure 8g,h,i). In WT mice examined during acute disease, we observed minimal Oil-Red-O staining (n = 9). By contrast, in the Axl-/- mice (n = 12) the Iba1+ and MAC3+ inflammatory cells present at the lesion site did not form a tight concentric ring and extensive Oil-Red-O+ debris was apparent.

#### There is more axonal damage in Axl-/- mice

**Figure 9:**
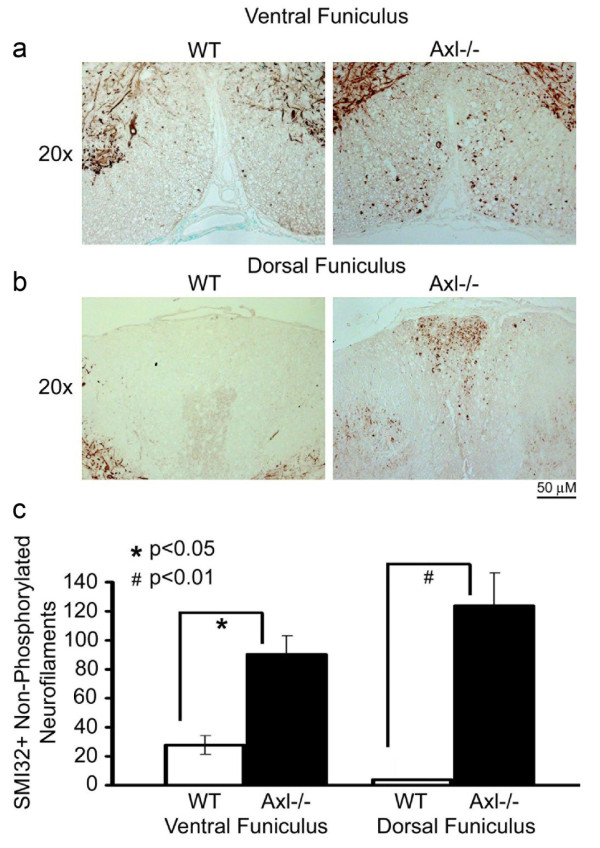
**The spinal cord of Axl-/- mice has more axonal damage in the ventral and dorsal funiculi than WT mice**. Seven micron paraffin-embedded WT and Axl-/- spinal cord sections were stained with SMI32, a mAb for non-phosphorylated neurofilaments. Representative 20× images are shown for the ventral and dorsal funiculi of WT and Axl-/- mice. b) SMI32+ non-phosphorylated neurofilament deposits were counted in the ventral and dorsal funiculi of three WT and three Axl-/- mice. Three or four sections were counted for each mouse and averaged. A student's t-test was performed to test significance; * = p < 0.05, # = p < 0.01.

SMI32 staining of non-phosphorylated neurofilaments is indicative of axonal damage in spinal cord white matter. In MOG-induced EAE, SMI32 staining is associated with lesions, and with swollen, severed or dying axons. A comparison between WT and Axl-/- mice showed significantly more SMI32+ axons in Axl-/- spinal cord than in WT spinal cord, especially in the dorsal (WT: 3.9 ± 0.9; Axl-/-: 123.6 ± 22.6; p < 0.01) and ventral (WT: 27.7 ± 6.3; Axl-/-: 90.1 ± 12.9; p < 0.05) funiculi (Figure 9).

Thus, our data summarized in Table 1 show that relative to WT lumbar spinal cord, the focal lesions in lumbar spinal cord of Axl-/- mice were larger, with fewer Iba1+ activated microglia/macrophages, a higher ratio of CD3+ T cells to Iba1+ activated microglia/macrophages, more Oil-Red-O+ debris, and more SMI32+ axons.

**Table 1:** Summary of clinical and pathological data for the acute phase of EAE in WT and Axl-/- mice

	WT	Axl-/-
Average size of lesions (p < 0.01)	0.13 ± 0.03	0.23 ± 0.02

Iba1+ microglia/macrophages per lesion (p < 0.01)	50.3 ± 4.7	24.2 ± 1.6

Ratio of CD3+ to Iba1+ cells per lesion (p < 0.01)	1.3 ± 0.07	3.7 ± 0.26

Extent of Oil-Red-O+ debris per lesion (p < 0.01)	+	+++

Mean number SMI32+ axons		
Ventral lumbar spinal cord (p < 0.05)	27.7 ± 6.3	90.1 ± 12.6
Dorsal lumbar spinal cord (p < 0.01)	3.9 ± 0.9	123.6 ± 22.6

## Discussion

EAE is an immune-mediated inflammatory disease of the CNS that results in myelin and CNS cell loss, and axonal damage. In this study, we used the MOG-induced mouse model of EAE to determine the contribution of Axl to inflammation in the CNS, including assessment of the role of Axl in cytokine/chemokine expression, microglia/macrophage migration and the efficiency with which these cells clear tissue debris. Based on the multiple functions of Axl pertinent to protection against EAE, results from our previous cuprizone study [15], and current data that showed Axl is upregulated in EAE, we predicted that Axl-/- mice would have a more severe clinical disease course and more severe pathology than WT mice. Consistent with this prediction, the results showed that although the day of disease onset was the same for Axl-/- and WT mice, Axl-/- mice had higher clinical indices at 14 days post-MOG-injection and that increased disease severity persisted in Axl-/- mice throughout the acute phase. In contrast to Axl-/- mice, the clinical course of MOG-induced EAE in Tyro3-/- and WT mice was the same, perhaps reflecting the upregulation of the mature 140 kDa form of Axl that compensated for the lack of Tyro3.

The acute phase of the disease is attributed to breakdown of the BBB, edema, and influx of inflammatory cells, resulting in myelin loss, and axonal damage in the spinal cord. The detection of higher clinical scores in the Axl-/- mice predicted there would be more damage in the spinal cord of Axl-/- mice than WT mice. As predicted, relative to WT mice the loss of Axl resulted in larger inflammatory cuffs comprised of CD45 and CD3 stained leukocytes but fewer lesion-associated Iba1+ microglia/macrophages. Spinal cord lesions in Axl-/- mice also displayed increased loss of myelin, increased accumulation of myelin debris, and significantly more SMI32+ axons. This increased axonal damage likely reflects increased loss of myelin, which renders axons more vulnerable to damage and axonal die-back [40,41].

The severity of disease is mediated at least in part by the increase in pro-inflammatory cytokines and chemokines. For example, an increase in TNFα can lead to increased vascular permeability and expression of chemoattractant cytokines, such as MCP1 and RANTES, which together could result in greater tissue damage in the Axl-/- spinal cord [42-44]. Previous studies have shown that RANTES is activated at the onset of EAE and that expression correlates with leukocyte accumulation in the CNS [45]. Activated astrocytes and cells of monocyte/macrophage lineage secrete MCP1, which binds to its ligand CCR2 to recruit other monocytes/macrophages to areas of damage. Naïve mice had very low levels of TNFα, MCP1, and RANTES mRNA and there was no difference in the lumbar spinal cord between Axl-/- and WT mice. However, during EAE, mRNA for these cytokines and chemokines were upregulated in the spinal cord with levels corresponding to the severity of disease. Flow cytometric analysis determined there was no difference in TNFα expression by infiltrating T cells in the brain and spinal cord between Axl-/- and WT mice. Therefore, the observed differences in TNFα mRNA in the lumbar spinal cord can likely be attributed to macrophages and/or resident CNS cells. Differences in cytokine expression in Axl-/- mice versus WT mice were most obvious in mice with lower CI's. At CI's ≤ 2 there was significantly more mRNA for TNFα and RANTES in the spinal cord of Axl-/- mice. At CI's > 2 no difference in the ratio of TNFα, MCP1, and RANTES transcripts was detected suggesting that the cell machinery was maximally producing these transcripts. Despite the increase in MCP1 transcripts in Axl-/- spinal cord during EAE, Iba1+ microglia/macrophages were under-represented in lesions relative to spinal cord lesions of WT mice. This may reflect a role for Axl in cell migration.

The most striking difference in spinal cord pathology between Axl-/- mice and WT mice was noted in the distribution of activated microglia associated with lesions and the lack of clearance of Oil-Red-O+ debris. Normally, resting microglia are evenly distributed throughout the gray and white matter of the spinal cord where they are thought to function as monitors of tissue damage and the influx of foreign material [46]. Once damage or a foreign antigen is detected microglia become activated and migrate to affected areas of the CNS [47]. Several studies have implicated a role for Axl signaling in cell migration. In studies of gliomas, dominant-negative Axl lacking the intracellular domain interfered with cell migration [48], and in GN11 cells Gas6 signaling downstream of Axl was implicated in cell migration [49]. We observed that although there was no difference in the number of microglia in the brain and spinal cord of mice with EAE, Axl-/- mice failed to display equivalent numbers of activated microglia/macrophages surrounding lesioned areas of the spinal cord when compared with WT counterparts. Furthermore, the microglia/macrophages recruited to lesioned areas of the cord in the WT mice appeared to form concentric rings around the lesions, suggesting that these cells acted to cordon off the damaged tissue. Failure to do this in the Axl-/- mice could account for the increase in lesion size noted in these mice. Failure to efficiently recruit phagocytic microglia has also been implicated in the reduced capacity of older animals to effectively remyelinate (reviewed in [25,50]). The additional Oil-Red-O+ debris associated with lesions in Axl-/- relative to lesions in WT mice is indicative of the persistence of myelin debris not cleared by Iba1+ or Mac3+ inflammatory cells. Several possibilities might account for the persistent Oil-Red-O+ debris including the increased clinical severity and higher scores observed in the Axl-/- mice resulting in extensive demyelination, the inability of the Axl-/- microglia/macrophages to efficiently migrate to the site of injury and/or inefficient uptake of the Oil-Red-O+ myelin debris by the inflammatory cells. In collaboration with the laboratory of Dr. Dianne Cox at Albert Einstein College of Medicine, *in vitro *uptake assays were performed to determine whether macrophages isolated from WT and Axl-/- mice efficiently engulfed debris. Qualitatively, there was no difference in FcγR-mediated or CR3-mediated phagocytosis as determined by equivalent uptake of IgG-coated erythrocytes or zymosan particles, respectively, in WT and Axl-/- macrophages (data not shown) indicating that macrophages from Axl-/-mice were capable of efficient engulfment.

In most animal models of CNS demyelination, elimination of myelin debris is a necessary prerequisite for remyelination and repair. In young animals this is accomplished efficiently by phagocytic macrophages and microglia (reviewed in [50]). Axl, Tyro3 and Mer have all been shown to be required for efficient phagocytosis of apoptotic cells [30]. Also, it is possible for myelin debris to persist in the lesion area as shown by extracytoplasmic Oil-Red-O staining observed in pathologic and necrotic tissue, a condition present in EAE lesions [51-53]. Thus, increased Oil-Red-O+ debris in Axl-/- mice during EAE likely reflects a failure of Iba1+ and/or MAC3+ inflammatory cells to efficiently migrate to the injury site in order to clear debris, leading to impaired remyelination and tissue damage.

## Conclusions

Taken together, these data support the conclusion that exacerbation of acute EAE in Axl-/- mice reflects the role of Axl in regulating the activity of cells of the monocyte/macrophage series, whilst having little effect on the activity of lymphocytes. This would be consistent with the known distribution of Axl, and other TAM receptors, which are constitutively but variably expressed by all subpopulations of monocytes/macrophages, but are not expressed by most mature lymphocytes (reviewed in [54]). The data show that Axl is important for several aspects of tissue homeostasis in the spinal cord of mice with EAE including migration of macrophages/microglia to sites of tissue damage, the efficient walling off of the lesion and the clearance of tissue debris that in turn likely limits the extent of damage. These findings have significant relevance for our understanding of the pathogenesis of MS. Our previous work has shown that Axl and a deleterious soluble form of Axl are upregulated in MS lesions, and that the soluble form of Axl negatively correlates with expression of the TAM receptor ligand Gas6 [5]. The data further suggest that increasing the availability of Gas6 in MS lesions may lead to enhanced Axl activation resulting in the arrest of lesion enlargement thus favoring remyelination and repair of tissue damage.

## Competing interests

The funding agency had no role in study design, data collection and analysis, decision to publish or preparation of the manuscript. The authors have no financial or non-financial competing interests to declare.

## Authors' contributions

JGW, CFB, VT and BSZ induced EAE in the mice. JGW and BSZ performed protein isolation and analysis (Western blot), immunohistochemistry, and Oil-Red-O staining. JGW and OL isolated RNA and performed quantitative PCR. MFG and FM did CNS preparations and isolated inflammatory cells for flow cytometry and analysis. MFG performed intracellular cytokine staining. The *ex vivo *proliferation assay was performed by CFB and BSZ. HAA performed the serum protein analysis. ALP supplied the Axl polyclonal antibody. All authors have read and approved the final version of the manuscript.

## References

[B1] Camenisch TD Koller BH Earp HS Matsushima GK A novel receptor tyrosine kinase, Mer, inhibits TNF-alpha production and lipopolysaccharide-induced endotoxic shock J Immunol 1999 162 3498 3503 10092806

[B2] Lu Q Lemke G Homeostatic regulation of the immune system by receptor tyrosine kinases of the Tyro 3 family Science 2001 293 306 311 10.1126/science.1061663 11452127

[B3] Scott RS McMahon EJ Pop SM Reap EA Caricchio R Cohen PL Earp HS Matsushima GK Phagocytosis and clearance of apoptotic cells is mediated by MER Nature 2001 411 207 211 10.1038/35075603 11346799

[B4] Binder MD Cate HS Prieto AL Kemper D Butzkueven H Gresle MM Cipriani T Jokubaitis VG Carmeliet P Kilpatrick TJ Gas6 deficiency increases oligodendrocyte loss and microglial activation in response to cuprizone-induced demyelination J Neurosci 2008 28 5195 5206 10.1523/JNEUROSCI.1180-08.2008 18480276 PMC3844801

[B5] Weinger JG Omari KM Marsden K Raine CS Shafit-Zagardo B Up-regulation of soluble Axl and Mer receptor tyrosine kinases negatively correlates with Gas6 in established multiple sclerosis lesions Am J Pathol 2009 175 283 293 10.2353/ajpath.2009.080807 19541935 PMC2708814

[B6] Janssen JW Schulz AS Steenvoorden AC Schmidberger M Strehl S Ambros PF Bartram CR A novel putative tyrosine kinase receptor with oncogenic potential Oncogene 1991 6 2113 2120 1834974

[B7] O'Bryan JP Frye RA Cogswell PC Neubauer A Kitch B Prokop C Espinosa R Le Beau MM Earp HS Liu ET axl, a transforming gene isolated from primary human myeloid leukemia cells, encodes a novel receptor tyrosine kinase Mol Cell Biol 1991 11 5016 5031 1656220 10.1128/mcb.11.10.5016-5031.1991 PMC361494

[B8] Graham DK Dawson TL Mullaney DL Snodgrass HR Earp HS Cloning and mRNA expression analysis of a novel human protooncogene, c-mer Cell Growth Differ 1994 5 647 657 8086340

[B9] Jia R Hanafusa H The proto-oncogene of v-eyk (v-ryk) is a novel receptor-type protein tyrosine kinase with extracellular Ig/GN-III domains J Biol Chem 1994 269 1839 1844 7507487

[B10] Funakoshi H Yonemasu T Nakano T Matumoto K Nakamura T Identification of Gas6, a putative ligand for Sky and Axl receptor tyrosine kinases, as a novel neurotrophic factor for hippocampal neurons J Neurosci Res 2002 68 150 160 10.1002/jnr.10211 11948660

[B11] Shankar SL O'Guin K Cammer M McMorris FA Stitt TN Basch RS Varnum B Shafit-Zagardo B The growth arrest-specific gene product Gas6 promotes the survival of human oligodendrocytes via a phosphatidylinositol 3-kinase-dependent pathway J Neurosci 2003 23 4208 4218 12764109 10.1523/JNEUROSCI.23-10-04208.2003 PMC6741089

[B12] Sainaghi PP Collimedaglia L Alciato F Leone MA Puta E Naldi P Castello L Monaco F Avanzi GC Elevation of Gas6 protein concentration in cerebrospinal fluid of patients with chronic inflammatory demyelinating polyneuropathy (CIDP) J Neurol Sci 2008 269 138 142 10.1016/j.jns.2008.01.005 18279894

[B13] Manfioletti G Brancolini C Avanzi G Schneider C The protein encoded by a growth arrest-specific gene (gas6) is a new member of the vitamin K-dependent proteins related to protein S, a negative coregulator in the blood coagulation cascade Mol Cell Biol 1993 13 4976 4985 8336730 10.1128/mcb.13.8.4976-4985.1993 PMC360142

[B14] Prieto AL Weber JL Tracy S Heeb MJ Lai C Gas6, a ligand for the receptor protein-tyrosine kinase Tyro-3, is widely expressed in the central nervous system Brain Res 1999 816 646 661 10.1016/S0006-8993(98)01159-7 9878891

[B15] Hoehn HJ Kress Y Sohn A Brosnan CF Bourdon S Shafit-Zagardo B Axl-/- mice have delayed recovery and prolonged axonal damage following cuprizone toxicity Brain Res 2008 1240 1 11 18804096 10.1016/j.brainres.2008.08.076

[B16] Heide I Sokoll AC Henz BM Nagel S Kreissig K Grutzkau A Grabbe J Wittig B Neubauer A Regulation and possible function of axl expression in immature human mast cells Ann Hematol 1998 77 199 205 10.1007/s002770050443 9858144

[B17] Sharif MN Sosic D Rothlin CV Kelly E Lemke G Olson EN Ivashkiv LB Twist mediates suppression of inflammation by type I IFNs and Axl J Exp Med 2006 203 1891 1901 10.1084/jem.20051725 16831897 PMC2118370

[B18] Van Der Voorn P Tekstra J Beelen RH Tensen CP Van Der Valk P De Groot CJ Expression of MCP-1 by reactive astrocytes in demyelinating multiple sclerosis lesions Am J Pathol 1999 154 45 51 10.1016/S0002-9440(10)65249-2 9916917 PMC1853444

[B19] Cannella B Raine CS The adhesion molecule and cytokine profile of multiple sclerosis lesions Ann Neurol 1995 37 424 435 10.1002/ana.410370404 7536402

[B20] Hofman FM Hinton DR Johnson K Merrill JE Tumor necrosis factor identified in multiple sclerosis brain J Exp Med 1989 170 607 612 10.1084/jem.170.2.607 2754393 PMC2189402

[B21] Selmaj K Raine CS Cannella B Brosnan CF Identification of lymphotoxin and tumor necrosis factor in multiple sclerosis lesions J Clin Invest 1991 87 949 954 10.1172/JCI115102 1999503 PMC329886

[B22] dos Santos AC Barsante MM Arantes RM Bernard CC Teixeira MM Carvalho-Tavares J CCL2 and CCL5 mediate leukocyte adhesion in experimental autoimmune encephalomyelitis--an intravital microscopy study J Neuroimmunol 2005 162 122 129 10.1016/j.jneuroim.2005.01.020 15833367

[B23] Rieckmann P Albrecht M Kitze B Weber T Tumani H Broocks A Luer W Helwig A Poser S Tumor necrosis factor-alpha messenger RNA expression in patients with relapsing-remitting multiple sclerosis is associated with disease activity Ann Neurol 1995 37 82 88 10.1002/ana.410370115 7818262

[B24] Tanuma N Abe S Shin T Kojima T Ishihara Y Arai Y Toyoshima S Matsumoto Y Pretreatment with T cell receptor peptides using a conventional immunization protocol does not induce effective protection against autoimmune encephalomyelitis Cell Immunol 1996 168 85 90 10.1006/cimm.1996.0052 8599843

[B25] Neumann H Kotter MR Franklin RJ Debris clearance by microglia: an essential link between degeneration and regeneration Brain 2009 132 288 295 18567623 10.1093/brain/awn109 PMC2640215

[B26] Rappert A Bechmann I Pivneva T Mahlo J Biber K Nolte C Kovac AD Gerard C Boddeke HW Nitsch R Kettenmann H CXCR3-dependent microglial recruitment is essential for dendrite loss after brain lesion J Neurosci 2004 24 8500 8509 10.1523/JNEUROSCI.2451-04.2004 15456824 PMC6729901

[B27] Streit WJ Conde JR Fendrick SE Flanary BE Mariani CL Role of microglia in the central nervous system's immune response Neurol Res 2005 27 685 691 16197805 10.1179/016164105X49463a

[B28] Hanisch UK Kettenmann H Microglia: active sensor and versatile effector cells in the normal and pathologic brain Nat Neurosci 2007 10 1387 1394 10.1038/nn1997 17965659

[B29] Heneka MT O'Banion MK Terwel D Kummer MP Neuroinflammatory processes in Alzheimer's disease J Neural Transm 2010 117 919 947 10.1007/s00702-010-0438-z 20632195

[B30] Seitz HM Camenisch TD Lemke G Earp HS Matsushima GK Macrophages and dendritic cells use different Axl/Mertk/Tyro3 receptors in clearance of apoptotic cells J Immunol 2007 178 5635 5642 17442946 10.4049/jimmunol.178.9.5635

[B31] Pluchino S Quattrini A Brambilla E Gritti A Salani G Dina G Galli R Del Carro U Amadio S Bergami A Furlan R Comi G Cescovi AL Martino G Injection of adult neurospheres induces recovery in a chronic model of multiple sclerosis Nature 2003 422 688 694 10.1038/nature01552 12700753

[B32] Laemmli UK Cleavage of structural proteins during the assembly of the head of bacteriophage T4 Nature 1970 227 680 685 10.1038/227680a0 5432063

[B33] Towbin H Staehelin T Gordon J Electrophoretic transfer of proteins from polyacrylamide gels to nitrocellulose sheets: procedure and some applications Proc Natl Acad Sci USA 1979 76 4350 4354 10.1073/pnas.76.9.4350 388439 PMC411572

[B34] Zamora-Leon SP Bresnick A Backer JM Shafit-Zagardo B Fyn phosphorylates human MAP-2c on tyrosine 67 J Biol Chem 2005 280 1962 1970 15536091 10.1074/jbc.M411380200

[B35] Chen L Brosnan CF Exacerbation of experimental autoimmune encephalomyelitis in P2X7R-/- mice: evidence for loss of apoptotic activity in lymphocytes J Immunol 2006 176 3115 3126 16493071 10.4049/jimmunol.176.5.3115

[B36] Bertenshaw GP Yip P Seshaiah P Zhao J Chen TH Wiggins WS Mapes JP Mansfield BC Multianalyte profiling of serum antigens and autoimmune and infectious disease molecules to identify biomarkers dysregulated in epithelial ovarian cancer Cancer Epidemiol Biomarkers Prev 2008 17 2872 2881 10.1158/1055-9965.EPI-08-0464 18843033

[B37] Venkataswamy MM Baena A Goldberg MF Bricard G Im JS Chan J Reddington F Besra GS Jacobs WR Jr Porcelli SA Incorporation of NKT cell-activating glycolipids enhances immunogenicity and vaccine efficacy of Mycobacterium bovis bacillus Calmette-Guerin J Immunol 2009 183 1644 1656 10.4049/jimmunol.0900858 19620317 PMC2719834

[B38] Mueller KL Hunter LA Ethier SP Boerner JL Met and c-Src cooperate to compensate for loss of epidermal growth factor receptor kinase activity in breast cancer cells Cancer Res 2008 68 3314 3322 10.1158/0008-5472.CAN-08-0132 18451158 PMC3878202

[B39] O'Bryan JP Fridell YW Koski R Varnum B Liu ET The transforming receptor tyrosine kinase, Axl, is post-translationally regulated by proteolytic cleavage J Biol Chem 1995 270 551 557 10.1074/jbc.270.2.551 7822279

[B40] Kuhlmann T Lingfeld G Bitsch A Schuchardt J Bruck W Acute axonal damage in multiple sclerosis is most extensive in early disease stages and decreases over time Brain 2002 125 2202 2212 10.1093/brain/awf235 12244078

[B41] Wujek JR Bjartmar C Richer E Ransohoff RM Yu M Tuohy VK Trapp BD Axon loss in the spinal cord determines permanent neurological disability in an animal model of multiple sclerosis J Neuropathol Exp Neurol 2002 61 23 32 11829341 10.1093/jnen/61.1.23

[B42] Ferrero E Zocchi MR Magni E Panzeri MC Curnis F Rugarli C Ferrero ME Corti A Roles of tumor necrosis factor p55 and p75 receptors in TNF-alpha-induced vascular permeability Am J Physiol Cell Physiol 2001 281 C1173 1179 11546653 10.1152/ajpcell.2001.281.4.C1173

[B43] Wolf G Aberle S Thaiss F Nelson PJ Krensky AM Neilson EG Stahl RA TNF alpha induces expression of the chemoattractant cytokine RANTES in cultured mouse mesangial cells Kidney Int 1993 44 795 804 10.1038/ki.1993.314 7505037

[B44] Chen YM Chiang WC Lin SL Wu KD Tsai TJ Hsieh BS Dual regulation of tumor necrosis factor-alpha-induced CCL2/monocyte chemoattractant protein-1 expression in vascular smooth muscle cells by nuclear factor-kappaB and activator protein-1: modulation by type III phosphodiesterase inhibition J Pharmacol Exp Ther 2004 309 978 986 10.1124/jpet.103.062620 14978197

[B45] Glabinski AR Tuohy VK Ransohoff RM Expression of chemokines RANTES, MIP-1alpha and GRO-alpha correlates with inflammation in acute experimental autoimmune encephalomyelitis Neuroimmunomodulation 1998 5 166 171 10.1159/000026333 9730682

[B46] Kreutzberg GW Microglia, the first line of defence in brain pathologies Arzneimittelforschung 1995 45 357 360 7763326

[B47] Gehrmann J Matsumoto Y Kreutzberg GW Microglia: intrinsic immuneffector cell of the brain Brain Res Brain Res Rev 1995 20 269 287 7550361 10.1016/0165-0173(94)00015-h

[B48] Vajkoczy P Knyazev P Kunkel A Capelle HH Behrndt S von Tengg-Kobligk H Kiessling F Eichelsbacher U Essig M Read TA Erber R Ullrich A Dominant-negative inhibition of the Axl receptor tyrosine kinase suppresses brain tumor cell growth and invasion and prolongs survival Proc Natl Acad Sci USA 2006 103 5799 5804 10.1073/pnas.0510923103 16585512 PMC1458653

[B49] Allen MP Linseman DA Udo H Xu M Schaack JB Varnum B Kandel ER Heidenreich KA Wierman ME Novel mechanism for gonadotropin-releasing hormone neuronal migration involving Gas6/Ark signaling to p38 mitogen-activated protein kinase Mol Cell Biol 2002 22 599 613 10.1128/MCB.22.2.599-613.2002 11756555 PMC139735

[B50] Franklin RJ Kotter MR The biology of CNS remyelination: the key to therapeutic advances J Neurol 2008 255 Suppl 1 19 25 18317673 10.1007/s00415-008-1004-6

[B51] Rémy C Fouilhé N Barba I Sam-Lai E Lahrech H Cucurella MG Izquierdo M Moreno A Ziegler A Massarelli R Décorps M Arús C Evidence that mobile lipids detected in rat brain glioma by 1H nuclear magnetic resonance correspond to lipid droplets Cancer Res 1997 57 407 414 9012466

[B52] Lahrech H Zoula S Farion R Rémy C Décorps M In vivo measurement of the size of lipid droplets in an intracerebral glioma in the rat Magn Reson Med 2001 45 409 414 10.1002/1522-2594(200103)45:3<409::AID-MRM1053>3.0.CO;2-O 11241697

[B53] Rizk T Montero-Menei C Jollivet C Benoit JP Menei P Pitfalls in the detection of lipid vectors in neural cell culture and in brain tissue J Biomed Mater Res A 2004 68 360 364 14704978 10.1002/jbm.a.20060

[B54] Lemke G Rothlin CV Immunobiology of the TAM receptors Nat Rev Immunol 2008 8 327 336 10.1038/nri2303 18421305 PMC2856445

